# Photophysiological and Photosynthetic Complex Changes during Iron Starvation in *Synechocystis sp.* PCC 6803 and *Synechococcus elongatus* PCC 7942

**DOI:** 10.1371/journal.pone.0059861

**Published:** 2013-03-19

**Authors:** Jared M. Fraser, Sarah E. Tulk, Jennifer A. Jeans, Douglas A. Campbell, Thomas S. Bibby, Amanda M. Cockshutt

**Affiliations:** 1 Department of Chemistry & Biochemistry, Mount Allison University, Sackville, New Brunswick, Canada; 2 School of Ocean and Earth Science, University of Southampton, National Oceanography Centre, Southampton, United Kingdom; Royal Netherlands Institute of Sea Research (NIOZ), The Netherlands

## Abstract

Iron is an essential component in many protein complexes involved in photosynthesis, but environmental iron availability is often low as oxidized forms of iron are insoluble in water. To adjust to low environmental iron levels, cyanobacteria undergo numerous changes to balance their iron budget and mitigate the physiological effects of iron depletion. We investigated changes in key protein abundances and photophysiological parameters in the model cyanobacteria *Synechococcus* PCC 7942 and *Synechocystis* PCC 6803 over a 120 hour time course of iron deprivation. The iron stress induced protein (IsiA) accumulated to high levels within 48 h of the onset of iron deprivation, reaching a molar ratio of ∼42 IsiA : Photosystem I in *Synechococcus* PCC 7942 and ∼12 IsiA : Photosystem I in *Synechocystis* PCC 6803. Concomitantly the iron-rich complexes Cytochrome b_6_f and Photosystem I declined in abundance, leading to a decrease in the Photosystem I : Photosystem II ratio. Chlorophyll fluorescence analyses showed a drop in electron transport per Photosystem II in *Synechococcus*, but not in *Synechocystis* after iron depletion. We found no evidence that the accumulated IsiA contributes to light capture by Photosystem II complexes.

## Introduction

We sought to determine the effects of iron depletion on photosynthetic physiology and allocations to the major thylakoid complexes in the model fresh water cyanobacteria *Synechococcus elongatus* PCC 7942 (hereafter *Synechococcus*) and *Synechocystis* sp. PCC 6803 (hereafter *Synechocystis*), whose photosynthetic and respiratory electron chains intersect ([1]–[3].

Iron is an essential component of multiple cellular structures, serving as a redox co-factor. The cyanobacterial photosynthetic system Photosystem I (PSI) and the intermediary electron transport complex cytochrome b_6_f both contain considerable amounts of iron and are thus synthesized in lower amounts during times of iron scarcity [4]–[6]. To adjust electron transport to these downstream constraints there is also often a decrease in Photosystem II (PSII) complexes under iron scarcity [6], [7]; this serves to lower the rate of electrons being introduced into the photosynthetic electron transport chain [5] so that PSI turnover does not limit PSII electron flow, thereby limiting production of dangerous oxygen radicals [7]. During iron depletion cyanobacteria accumulate an alternate chlorophyll binding complex, the iron stress induced protein (IsiA), or CP43′ [6], which can attach to PSI and increase its effective absorption cross section [8], partially offsetting the effects of having fewer PSI complexes [8], and/or storing chlorophyll in a down-regulated state with safe dissipation of excitation [9], [10].

There are numerous other adaptations in cyanobacteria in response to iron depletion. Like most bacteria they improve their ability to obtain iron through production of siderophores, which scavenge iron from the environment [11]. They may also replace some iron-containing proteins with functional analogs that contain no iron, including replacing ferredoxin with flavodoxin as the terminal electron acceptor of photosynthesis [6], [11]. Nitrogen-fixing cyanobacteria like *Trichodesmium* down-regulate expression of the iron-rich nitrogenase enzyme under conditions of iron limitation [12]–[14], before down-regulating their photosynthetic system. The major light harvesting phycobilisome complexes contain abundant amino acids and bilin chromophores whose synthesis relies upon iron-dependent metabolic paths [15], thus phycobilisome content declines under iron limitation [6], [16], probably as a consequence of secondary N-limitation induced by Fe-limitation.

When iron levels decline IsiA is expressed to compensate for the diminished light capture through its role as a chlorophyll a binding protein. IsiA accumulation can double the light harvesting capacity of PSI [17], [18] through binding to a PSI trimer, forming an IsiA-PSI supercomplex consisting of eighteen IsiA and three PSI complexes [19]. The PSI trimer is not necessary for complex formation as six IsiA complexes can bind to a single PSI complex [17]. One study claims that IsiA can form an inner ring of 12–14 units and an outer ring of 19–21 [20] units around a PSI monomer, and can even assemble into supercomplexes without PSI [21]. Further to its role as an antenna protein serving PSI, IsiA also has other possible functions which help the cell adapt to conditions of iron scarcity. IsiA may safely store chlorophyll to allow rapid re-addition to newly synthesized PSI after iron levels rise. IsiA-bound chlorophyll may absorb and dissipate light energy to protect the cell from oxidative stress [21]. IsiA has also been proposed to serve as a light capture antenna for PSII as it does for PSI, allowing for decreased numbers of higher functioning PSII complexes [22]. The induction of IsiA also parallels considerable changes in membrane lipid composition and the cellular content of photoprotective carotenoids [23].

We subjected two strains of model cyanobacteria to 120 hours of iron depletion to track their acclimation. We quantified key subunits mediating thylakoid photophosphorylation using immunoblotting. In parallel we used chlorophyll fluorescence measurements to track changes in PSII function, the functional absorbance cross section serving PSII photochemistry, and changes in electron transport during iron depletion.

## Materials and Methods

### Culturing and Sampling

We inoculated *Synechococcus* PCC 7942 or *Synechocystis* PCC 6803 stock cultures into 50 mL of BG-11 [24] buffered with 10 mM MOPS to pH 7.5 in acid washed, sterile culture tubes. The cultures were bubbled with air and grown under 60 to 70 μmol photons•m^−2^•s^−1^ at 33°C. 4 days after inoculation, 30 mL of culture was centrifuged (Beckman Coulter Avanti J-20 centrifuge) at 4500 rpm for 6 minutes (for *Synechococcus*) or 6500 rpm for 7 minutes (for *Synechocystis*) in 40 mL polypropylene tubes. The media supernatant was discarded and the cell pellet was re-suspended in approximately 30 mL BG-11 lacking added iron to wash residual iron off the cell pellets. The re-suspended cells were then re-centrifuged. The supernatant was discarded and the pellet was re-suspended in 30 mL of BG-11 lacking added iron media. The culture was then aseptically transferred to a 500 mL acid washed, sterile Erlenmeyer flask containing approximately 200 mL of BG-11 lacking added iron and buffered with 10 mM MOPS at pH 7.5. As before, the cultures were bubbled with air and grown at a light level between 60 and 70 μmol photons·m^−2^·s^−1^ at 33°C.

The time zero samples were taken from the remaining iron replete culture volume. Approximately 10 mL was added to a 40 mL centrifuge tube along with 5 μL of 10% pluronic acid (Invitrogen, Cat. No. 24040-032) and centrifuged at 4500 rpm for 6 minutes. The supernatant was discarded until there was 1-2 mL remaining. The remaining medium was used to re-suspend the pellet, which was then transferred to a 2 mL microfuge tube (Progene, Cat. No. 24-MCT-200-CS) and centrifuged for 10 minutes at 14,000 rpm in a microfuge (Hettich Mikro 20). The supernatant was discarded and the cell pellet stored at −80°C. Samples from the iron depleted cultures were collected in a similar manner after 0, 48, 96, and 120 consecutive hours.

### Spectrophotometry

Liquid samples of about 1 mL of culture were placed into cuvettes each day of the iron depletion trial for measurements using a spectrophotometer (Shimadzu UV1800). An absorbance spectrum was taken from 350–750 nm. Absorbance at 750 nm and absorbance peaks around 680 nm (chlorophyll) and 630 nm (phycobilisomes) were measured using Shimadzu UV Probe software.

### Fluorometer Measurements

Liquid samples were taken for measurements in a fluorometer (PSI FL 3500) using the Fast Repetition Rate fluorescence technique [25]. A series of 40 rapid repetitions of 2 μs flashes of either red-orange (625 nm; ∼30,000 μmol photons m^−2^ s^−1^) or blue (455 nm; ∼100,000 μmol photons m^−2^ s^−1^) light separated by 2 μs of darkness were used to progressively close PSII reaction centres. We chose these wavelengths to preferentially excite phycobilisome pigments (at 625 nm) or chlorophyll (at 455 nm). The complete flash series took only 160 μs, while it takes ∼1000 μs for a closed PSII to pass an electron into electron transport. Thus a closed PSII could not reopen during the measurements, which led to fluorescence increasing with each flashlet. The PSI fluorometer system we used includes an emission filter combination of an HP690, passing light greater than 690 nm; an RG695, passing light greater than 695 nm and an LP720 passing light less than 720 nm to monitor fluorescence emission in the 695 to 720 nm waveband. After the initial flash series we activated actinic light using both red and blue sources simultaneously at a range of levels, and re-captured fluorescence rise curves to assess PSII function in illuminated cells. These fluorescence rise curves were analyzed using the PSIWORX script for MATLAB written by Audrey Barnett and published on sourceforge.net, to extract the functional absorbance cross section serving PSII photochemistry (σ_PSII_; A^2^•quanta^−1^) for either red-orange (625 nm) or for blue (455 nm) light; the maximum quantum yield for PSII (F_V_/F_M_); the quantum yield for electron transport for open PSII centers (ΦPSII) and functional absorbance cross section serving PSII photochemistry (σ_PSII_'; A^2^ ·quanta^−1^) for PSII centers still open in cells under illumination.

We estimated the electron transport rate away from PSII following Huot & Babin [26] as:

PSII electron transport  =  σ_PSII_' x I x q_P_,

where, σ_PSII_' (A^2^ ·quanta^−1^) is the effective absorbance cross section serving PSII photochemistry at the given light level I (photons·A^−2^·s^−1^) and q_P_ (F_M_'-F_S_)/(F_M_'-F_O_') is the proportion of PSII instantaneously open and ready to perform photochemistry under light level I [27]. Our single-turnover measures of chlorophyll fluorescence gave us direct measures of F_M_', the maximal fluorescence level with all PSII centers closed under a given illuminated state, and F_S_, the steady state fluorescence level under a given illuminated state. F_O_' is the baseline fluorescence level with PSII open but with the cell in a state of acclimation to a given illumination. In these strains F_O_' is similar to F_O_, baseline fluorescence measured from dark acclimated cells [28]. We therefore approximated, F_O_' using our measured F_O_ from cells in the dark.

We then multiplied PSII electron transport by the content of PSII, approximated as fmol PsbA•μg protein^−1^, valid for cells growing under low to moderate light [29] and (Shaver & Campbell, unpub.). The resulting estimate of PSII electron transport per total protein reflects both changes in PSII performance and changes in PSII content, which were considerable over the course of the iron depletion experiment.

### Protein Extraction and Immunoquantitations

Cell pellets were taken from the freezer and the approximate pellet size was determined to the nearest 25 μL. 1X extraction buffer was made using 50X Pefabloc SC serine protease inhibitor (Roche Applied Science) and previously prepared 4X extraction buffer containing 0.55 M TRIS buffer, 0.3 M LDS, 4.3 M glycerol, and 2 mM EDTA [30]. The pellet was re-suspended in approximately 200 μL of 1X extraction per 25 μL of pellet and transferred to a FastPrep lysing tube (MP Bio FastPrep Lysing Matrix D Tubes). The tubes were agitated in a FastPrep (MP Bio FastPrep-24) for 3 cycles lasting 1 minute, with 1 minute on ice between cycles. The samples were then centrifuged in a microcentrifuge and the supernatant transferred to a 1.5 mL microfuge tube.

Total protein was quantified using the Bio-Rad DC protein assay kit with the included Bovine Gamma Globulin protein standards of known concentrations. 5 μL of sample were placed into a pre-read 96-well plate with 25 μL of reagent A' and 200 μL of reagent B. After 15 minutes on a rotating table, the absorbance at 750 nm was measured for each well using a Versamax microplate reader. The software Softmax Pro was used to create a standard curve and determine the content of protein.

Protein extract samples were prepared with 4X LDS sample buffer (Invitrogen, Cat. No. NP0007) diluted to 1X with ddiH_2_O. Dithiothreitol (DTT) (Invitrogen, Cat. No. D-1532) was added to the samples to give a final concentration of 50 mM. Protein extract samples were prepared so that either 1 μg (for PsbA, PsbD in both strains, and AtpB in 6803) or 3 μg (for IsiA, PetC, PsaC in both strains, AtpB in 7942) of total protein was added into each gel lane. Standards (obtained from Agrisera AB, Sweden) were prepared in a similar fashion to concentrations appropriate for each protein. Samples and standards were heated for 5 minutes at 70°C.

The day before electrophoresis, 700 mL of 1X MES running buffer was prepared from 20X MES Running Buffer (Invitrogen, Cat. No. NP0002). 500 μL of 0.5 M DTT was added to 200 mL of MES. Running buffer with and without DTT was cooled overnight at 4°C to prevent overheating during electrophoresis.

For electrophoresis, a Novex XCell SureLock Mini Cell (Invitrogen, Cat. No. EI0001) was set up and 2 15-well 4–12% gradient Bis-Tris NuPAGE gels (Invitrogen, Cat. No. NP03321BOX) were placed inside. The 1X MES running buffer with DTT was poured into the inner chamber, and the outer chamber was filled ¾ full with 1X MES running buffer. Samples were loaded into the gel along with standards and a molecular marker made up of 4 μL Novex Sharp pre-stained protein standard (Invitrogen, Cat. No. LC5800) and 0.4 μL Magic Mark XP Western Standard (Invitrogen, Cat. No. LC5603). Gels were electrophoresed for 40 minutes at 200 V.

During electrophoresis, 1X transfer buffer was prepared from NuPAGE 20X transfer buffer (Invitrogen, Cat. No. NP006). For each gel, 2 filter papers and 2 sponges were soaked in 1X transfer buffer. One polyvinylidene fluoride (PVDF) membrane for each gel was soaked in methanol, followed by soaking in 1X transfer buffer. Transfers were run in XCell II™ Blot Module Kit (Invitrogen, Cat. No. EI0001) with water surrounding the blotting cell for 80 minutes at 30 V for 2 gels, and 60 minutes at 30 V for 1 gel. Upon completion of transfer, membranes were incubated in 2% ECL Advance blocking reagent (GE Healthcare, Cat. No. RPN2135) and agitated on a rotating table for 1 hour. The blocking reagent was prepared with TBS-T (Tris buffered saline and 0.01% Tween 20 (Invitrogen, Cat. No. 003005)). After 1 hour, the blocking reagent was discarded. Primary antibody diluted in blocking reagent was added. All antibodies were obtained from Agrisera AB, Sweden. Dilutions used were: IsiA (AS06–111) 1:1,000; PetC (AS08-330) 1:10,000; PsaC (AS10-939) 1:5,000; AtpB (AS05–085) 1:25,000; PsbA (AS05–084) 1:50,000; PsbD (AS05–146) 1:50,000. After 1 hour, the membrane was washed in TBS-T twice briefly, followed by 1×15 minute wash and 3×5 minute washes with agitation on a rotator table. The membrane was then incubated with secondary antibody diluted in TBS-T for 1 hour. All secondary antibodies were goat anti-rabbit IgG (ImmunoReagents Inc, lot 14-122-042810) and were diluted to ½ the strength of the primary antibody to a maximum dilution of 1:50 000. The previous washing procedure was performed after the 1 hour incubation. 500 μL each of ECL Advance Solutions A and B (GE Healthcare, Cat. No. PRN2135) were mixed for each membrane to be imaged. The mixture was poured over the membrane, which was then incubated in the dark for 5 minutes and imaged using a Versa-Doc Imaging system (BioRad Cat. No. 1708030). Images were analyzed using Image Lab 3.0 software (BioRad) for quantification of proteins.

To estimate changes in chlorophyll allocations among the major chlorophyll-binding complexes over the course of iron depletion we followed the approach of Ryan-Keogh et al. [18]. Using the chlorophyll binding stoichiometries of the major complexes [31], [32] we multiplied IsiA subunit contents per μg of total cellular protein by 12 chlorophyll bound per IsiA monomer. We multiplied PsaC subunit contents per μg of total cellular protein, a proxy for the content of PSI complexes, by 100 chlorophyll bound per PSI monomer. We multiplied PsbA subunit contents per μg of total cellular protein, a proxy for PSII structural content in cultures growing under moderate light [29] and (Shaver & Campbell, unpub.) by 36 per PSII monomer. Using PsbD content as an alternate proxy for PSII content gave similar results (not presented).

## Results and Discussion

### Growth slows and pigmentation changes during iron depletion

**Figure 1:**
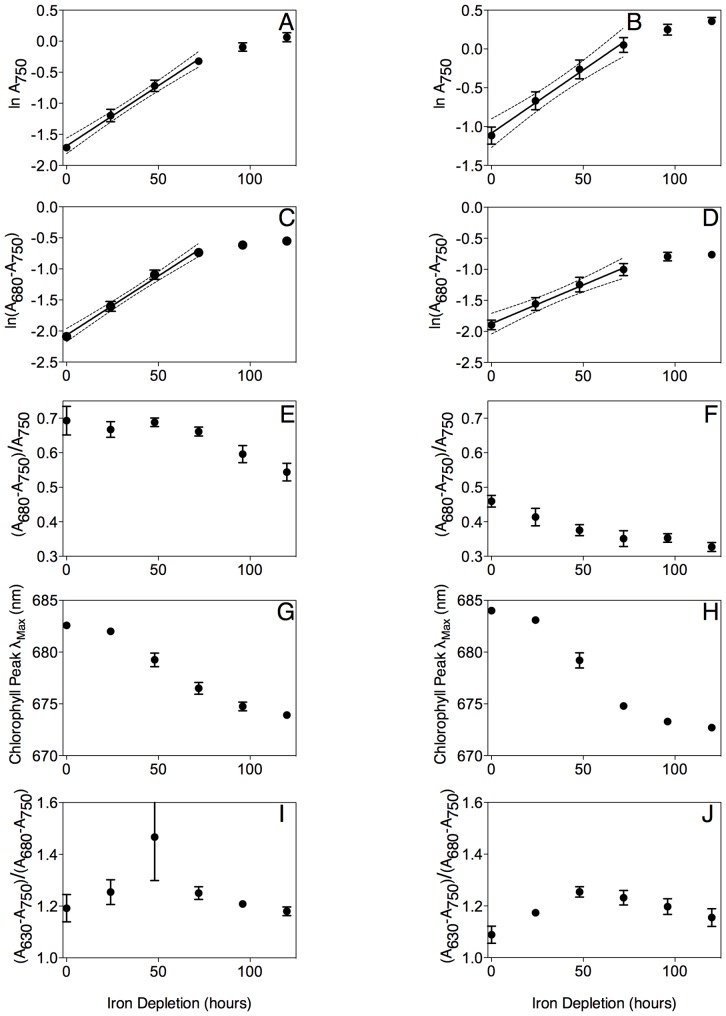
Spectral characteristics of *Synechococcus* (A, C, E, G, I) and *Synechocystis* (B, D, F, H, J) over a 120 hour iron depletion time course. Data were compiled from 6 (*Synechococcus*) or 5 (*Synechocystis*) replicate time course experiments. (A, B) ln A_750_ to track optical scattering, a proxy for culture cell suspension density. We fit measurements over the first 72 h with a linear regression to estimate the cell specific growth rates. Dotted lines show 95% confidence intervals on the slope of the regression; Data presented are mean +/− standard error, n = 5 or 6. (C, D) ln (A_680_–A_750_) to track chlorophyll content of the cultures. Dotted lines show 95% confidence intervals on the slope of the regression; Data presented are mean +/− standard error, n = 5 or 6. (E, F) (A_680_–A_750_)/(A_750_) to track chlorophyll per cell. (G, H) The wavelength for the chlorophyll absorbance peak, an optical measure of the accumulation of chlorophyll bound to IsiA. (I, J) (A_630_–A_750_)/(A_680_–A_750_) to track phycobilisome absorbance normalized to chlorophyll absorbance. Data presented are mean +/− standard error, n = 5 or 6.

**Figure 2:**
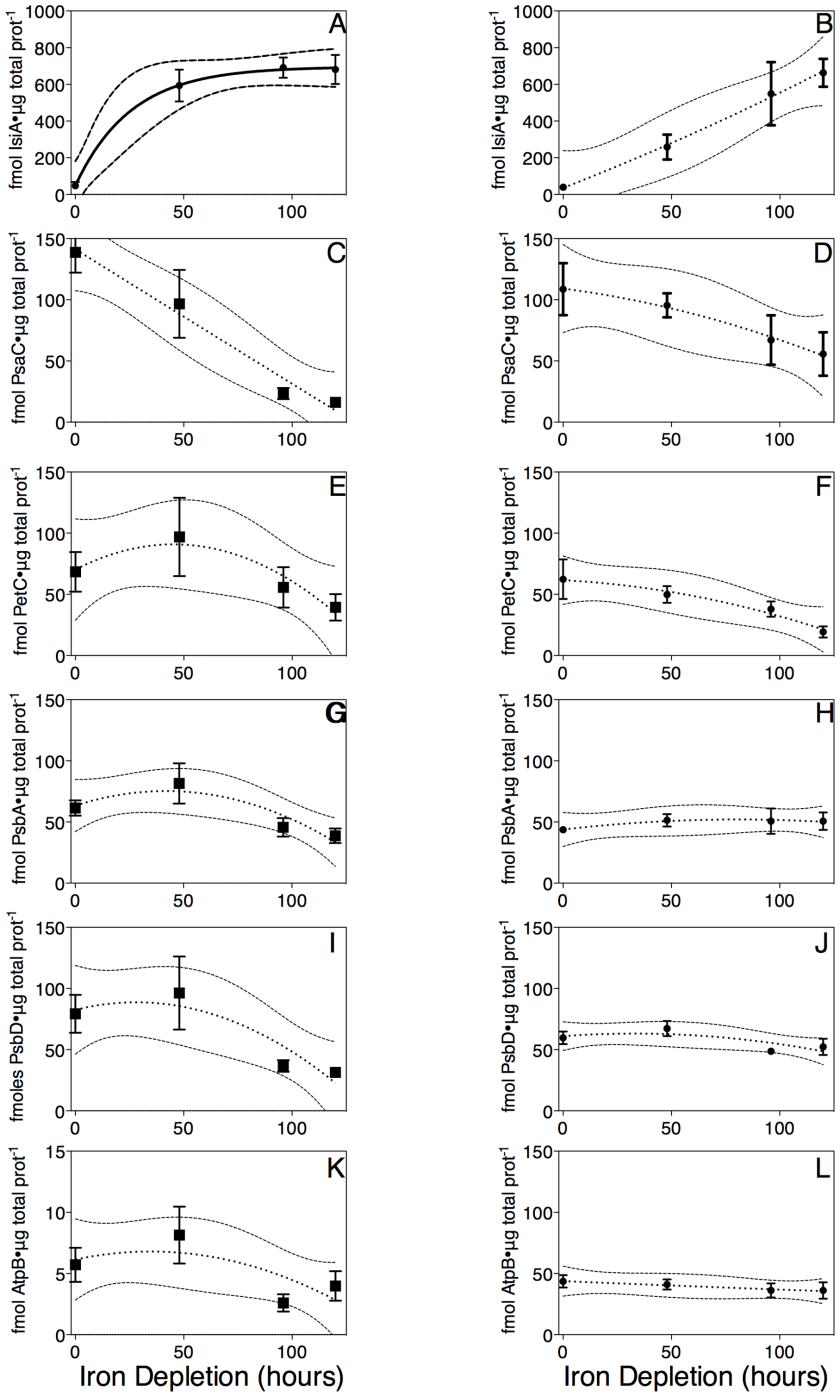
Content of key protein subunits in *Synechococcus* (A, C, E, G, I, K) and *Synechocystis* (B, D, F, H, J, L) over a 120 hour iron depletion time course. Data were compiled from 6 (*Synechococcus*) or 5 (*Synechocystis*) replicate immunoblots of cyanobacterial protein extracts from 6 (*Synechococcus*) or 5 (*Synechocystis*) replicate time course experiments (A, B) IsiA, (C, D) PsaC, (E, F) PetC,(G, H) PsbA, (I, J) PsbD, (K, L) AtpB. Protein subunit contents are expressed in femtomoles of protein per μg of total cellular protein. Data presented are mean +/− standard error, n = 5 or 6. Curve fits are second order polynomials with 95% confidence intervals plotted as outer dotted lines, except for Figure 2A which was fit with an logistic growth function, since IsiA reached a clear plateau.

Figure 1A and 1B present measurements of light scatter at A_750_, a proxy for cell suspension density, showing that both *Synechococcus* (0.019±0.001 h^−1^) and *Synechocystis* (0.016±0.002 h^−1^) maintained exponential growth over the first ∼72 h of iron depletion, but thereafter their growth slowed and dropped below the exponential trend line. The accumulation of chlorophyll, tracked as A_680_–A_750_, in the cell cultures followed a similar pattern in *Synechococcus* (Fig. 1C) (0.019±0.001 h^−1^), with a steady ratio of chlorophyll per cell over the first 72 h of iron depletion (Fig. 1E). In contrast, in *Synechocystis* the accumulation of chlorophyll was slower (Fig. 1D) (0.012±0.002 h^−1^), so that chlorophyll per cell was declining even over the first 72 hours of iron depletion (Fig. 1F). Over the iron depletion period the chlorophyll absorbance peak shifted to shorter wavelengths (Fig. 1G, F) [6], [33] with similar kinetics in both species as the major pool of chlorophyll-binding proteins shifted to IsiA (Fig. 2A, B) from PSI (Fig. 2C, D). In parallel with the changes in the chlorophyll pool the content of phycobilisome pigment, normalized to chlorophyll absorbance, started to decline after 48 h of iron depletion (Fig. 1I, J).

### IsiA accumulates during iron depletion

Iron stress induced protein (IsiA) has been purported to have several essential functions in cells under iron scarcity [8], [10], [18]. IsiA was maximally induced to 662 fmol/μg total protein within the first 48 hours of iron depletion in *Synechococcus* PCC 7942 (Fig. 2A; p<0.05 from 1 way ANOVA with Dunnett's Multiple Comparison Test). In *Synechocystis* IsiA induction (Fig. 2B) followed a fairly linear curve, reaching statistical significance by 96 h (p<0.05 from 1 way ANOVA with Dunnett's Multiple Comparison Test), and suggesting that IsiA levels would likely continue to increase beyond 120 hours of iron depletion in *Synechocystis*. The pattern and extent of IsiA accumulation in *Synechocystis* is similar to that seen by Ryan-Keogh et al. [18] despite their different culture growth conditions with inclusion of glucose in the medium, much lower growth light (10 μmol photons·m^−2^·s^−1^) and a 12:12 light dark cycle.

### Photosystem I levels decline during iron depletion

Photosystem I is responsible for catalyzing light induced charge separations, resulting in the transfer of electrons from plastocyanin to ferredoxin [34]. Each fully functional PSI complex contains 12 iron molecules, making it expensive to synthesize in terms of iron. Because of this, cyanobacteria accumulate less PSI during conditions of iron scarcity [4], [6]. We measured PsaC content, a subunit essential for PSI function, to track the quantity of PSI in each strain throughout the course of the iron depletion. In *Synechococcus*, PsaC levels declined significantly within 96 h of iron depletion (Fig. 2C; p<0.05 from 1 way ANOVA with Dunnett's Multiple Comparison Test), while in *Synechocystis* there was a smaller, slower decrease (Fig. 2D) that did not reach the threshold of statistical significance by 120 h (p>0.05 from 1 way ANOVA with Dunnett's Multiple Comparison Test). The smaller change in PSI content in *Synechocystis* could reflect slower induction of iron starvation relative to *Synechococcus*, indicated by the lack of a plateau in IsiA induction in *Synechocystis* (Fig. 2B). Although the change in the amount of PSI per ug protein in *Synechocystis* (a decrease to 50% of pre-starvation values) is similar to that reported by Sandstrom et al., [6] and Ryan-Keogh et al. [18], the absolute values reported here are approximately double those reported by Ryan-Keogh, likely reflecting the different culture growth conditions. If one assumes that *Synechocystis* was not fully iron starved at the end of the experiment, these results agree with the hypothesis that PSI levels drop during iron scarcity in an effort to conserve the cellular iron budget. This iron conservation mechanism can in turn cause oxidative stress because less PSI capacity is present to carry the electron flow from a highly reduced plastoquinone pool [7].

### Cytochrome b6f content declines slowly under iron depletion

Cytochrome b_6_f is the complex responsible for taking electrons from the plastoquinone pool and donating them to plastocyanin on their way to PSI in photosynthetic electron transport [35]. Because it is an iron-expensive complex to synthesize, in times of iron scarcity it is synthesized in much lower quantities [4]–[6]. In *Synechococcus* (Fig. 2E) the decline in Cytochrome b_6_f abundance did not reach the threshold of statistical significance below T0 levels within 120 h of iron depletion (p>0.05 from 1 way ANOVA with Dunnett's Multiple Comparison Test). In *Synechocystis* (Fig. 2F) the decline in Cytochrome b_6_f abundance reached statistical significance within 120 h of iron depletion (p<0.05 from 1 way ANOVA with Dunnett's Multiple Comparison Test). Although photosynthetic and respiratory electron transport intersect in both *Synechococcus* and in *Synechocystis*, there are important distinctions between the strains. Notably, the terminal respiratory cytochrome oxidase complex is localized predominantly to the cytoplasmic membrane in *Synechococcus* [1], but to the thylakoid membrane in *Synechocystis* [2]. The strain-specific patterns of decline in the intermediary Cytochrome b_6_f complex may relate to these distinctions in capacity to remove electrons from the thylakoid membrane. Unfortunately, at the time of this study, we did have in hand an antibody to track changes in the cytochrome oxidase complex itself.

### Photosystem II

Photosystem II is responsible for catalyzing the light induced splitting of water in photosynthesis [36]. The content of the PSII protein subunits PsbA (Fig. 2G) and PsbD (Fig. 2I) varied significantly over the 120 hours of iron depletion in *Synechococcus* (p<0.05 from 1 way ANOVA) but did not drop significantly below the T0 level (p>0.05 with Dunnett's Multiple Comparison Test). PsbA and PsbD content did not vary significantly in *Synechocystis* (Fig. 2H, J), perhaps because the onset of iron stress was slower in *Synechocystis*. It is important to recall that an active PSII repair cycle involves turnover of the PsbA and to an extent the PsbD (Fig. 2I, J) proteins in cyanobacteria [37], [38]. Therefore, the content of PSII protein subunits is not necessarily equivalent to the content of active PSII centers since at all times at least some of the PSII subunits are engaged in the PSII repair cycle.

### ATP Synthase

ATP Synthase utilizes the proton gradient generated by the electron transport chain to power the synthesis of ATP. In ATP synthase alternating α and β subunits arranged around a δ subunit form three catalytic nucleotide binding sites [39]. There are 3 α (AtpA) and 3 β (AtpB) subunits in each ATP Synthase complex. AtpB contents in *Synechococcus* (Fig. 2K) and *Synechocystis* (Fig. 2L) remained stable across the iron depletion time course. Interestingly, there was approximately 10 times more AtpB detected in *Synechocystis* than in *Synechococcus* at both the start and end of the experiment. If all AtpB is associated with ATP Synthase, these results indicate that there is as much as a tenfold difference between the amounts of ATP Synthase in the two strains.

### PSI/PSII

The high PSI:PSII ratio inherent to cyanobacteria [35] helps maximize electron transport away from PSII as there are multiple PSI complexes to carry electrons away from the plastoquinone pool for each PSII. The ratio of PsaC (PSI subunit) to PsbA (PSII subunit) was 2.3 in *Synechococcus* at the start of the iron starvation and similarly was 2.5 in *Synechocystis*. This indicates there are indeed multiple PSI for each PSII in both cyanobacterial species when iron is not limiting.

PSI levels dropped during this experiment in both *Synechococcus* and *Synechocystis*. Accordingly, the PSI:PSII ratio declined sharply in both strains during the iron depletion, to 0.4 in *Synechococcus* and to 1.1 in *Synechocystis*. Instead of having multiple PSI for every PSII, by the end of the iron starvation there were more PSII subunits than PSI subunits in the cell. To prevent the oxidative stress that would normally occur under these conditions the cell must be undergoing protective processes to limit over-reduction of the plastoquinone pool. The changes in PSI:PSII ratios presented here for *Synechocystis* are very similar to those reported by Ryan-Keogh [18] (reported as PSII:PSI) and Schrader et al. [16] despite differing growth conditions, while different from those reported here for *Synechococcus*, which decrease to 16% of pre-starvation values as opposed to 50% for *Synechocystis*, showing differences between the species in the onset of their acclimation to low iron conditions.

### Ratios of Cytochrome b_6_f to PSII and ATP Synthase to PSII

With the quantitative immunoblotting approach used in this study we followed the ratios of not only PSI:PSII but also Cytochrome b_6_f:PSII and ATP Synthase:PSII. *Synechococcus* showed little change in Cytochrome b_6_f:PSII ratios with pre-starvation values of 1.2 and a ratio of 1.0 after 5 days of iron depletion. In contrast, *Synechocystis* showed a marked and progressive decline in the ratio, with a pre-starvation value of 1.2 and a ratio of 0.3 following 5 days of iron depletion. It is interesting that the species that appeared to suffer the least iron stress, demonstrating less decrease in PSI and less accumulation of IsiA, showed the greatest loss of Cytochrome b_6_f relative to PSII.

The ratio of ATP Synthase:PSII was measured as the ratio of AtpB/(3 AtpB/ATP Synthase complex)(fmoles/ug protein)/PsbA (fmoles/ug protein). *Synechocystis* had a ratio of 0.46 ATP Synthase complexes (1.4 catalytic sites) per PSII complex in the pre-starvation growth conditions. Following 5 days of iron starvation this species had 0.23 ATP Synthase complexes (0.7 catalytic sites) per PSII complex, showing a modest drop in capacity to synthesize ATP. *Synechococcus* had markedly fewer ATP Synthase complexes per PSII, with 0.02 (0.05 catalytic sites) in the pre-starvation conditions and 0.03 (0.10 catalytic sites) after 5 days of iron starvation. We have repeatedly observed this low level of AtpB in *Synechococcus* in comparison to *Synechocystis* (Cockshutt et al., unpublished results). Peschek et al. [2] observed a similar difference in the ATPase activities measured from membrane preparations from these two species.

### IsiA:PSI

While the light capture functions of IsiA are contested [9], most studies find that it serves as an iron-inexpensive antenna for PSI [8] to increase the effective absorption cross section of the iron rich PSI complex, which helps the cell compensate for the lower levels of this complex present under iron scarce conditions [8], [18]. Crystal structures show that IsiA can associate with PSI trimers in an 18mer ring, giving a 6:1 molar ratio [19]. Crystal structures also show that a second ring can associate with the IsiA-PSI complex, resulting in a total ratio of 43 IsiA per PSI trimer, or about 14 IsiA per PSI [21]. *Synechococcus*, in this experiment, had a final IsiA:PSI molar ratio of 42, while *Synechocystis* had a final IsiA:PSI molar ratio of 12. These ratios, particularly in *Synechococcus*, are well in excess of the maximum ratio of IsiA known to associate with PSI in crystal structures. This suggests that under these growth conditions IsiA forms complexes without PSI, supporting a role in photoprotection, beyond acting as a PSI antenna [10], [21], [23].

### Does IsiA Contribute to PSII light capture?

**Figure 3:**
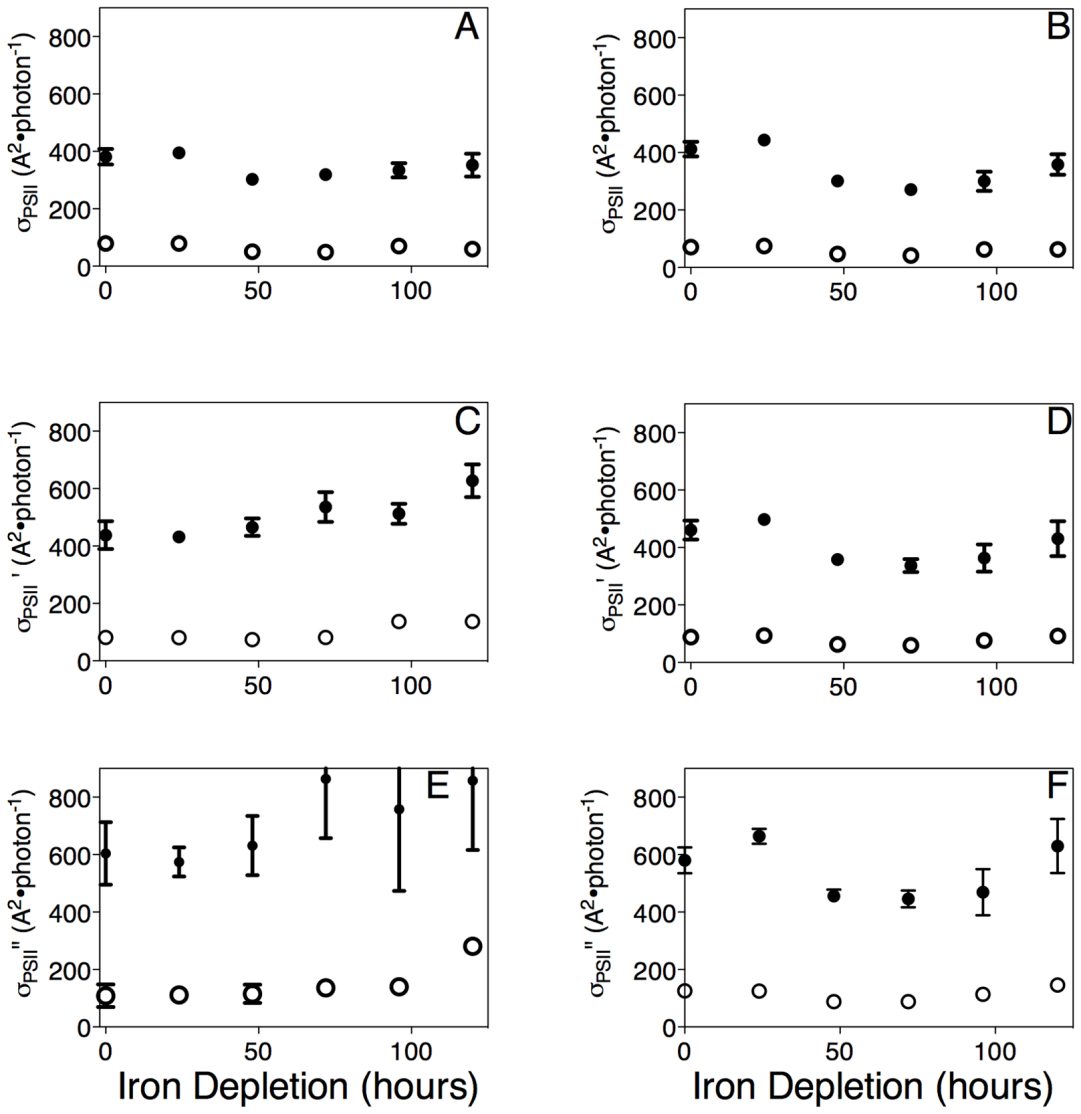
Functional absorption cross section serving PSII chemistry (σ_PSII_) in *Synechococcus* (A, C, E) and *Synechocystis* (B, D, F) over a 120 hour iron depletion timecourse. Closed symbols are σ_PSII_ for red light; open symbols are σ_PSII_ for blue light. (A, B) σ_PSII_ measured for all active PSII centers in cells, measured after 1 min dark acclimation. (C, D) σ_PSII_' measured for PSII centers remaining open in cells exposed to actinic light of 66 μmol photons·m^−2^·s^−1^ for 10 seconds before measurement. (E, F) σ_PSII_'' measured for PSII centers remaining open in cells under actinic light of 262 μmol photons·m^−2^·s^−1^for 10 seconds before measurement. Data were compiled from 6 (*Synechococcus*) or 5 (*Synechocystis*) replicate measurements from 6 or 5 separate cyanobacterial cultures. Data presented are mean +/− standard error, n = 5 or 6.

We determined the effective absorbance cross section serving photosystem II, or σ_PSII_, for each strain for both red-orange (625 nm) and blue (455) light (Fig. 3A, B). σ_PSII_ varies with wavelength because it is a function of both the light absorbance spectra of the pigments that comprise the PSII antenna, and the abundances of different pigment proteins in the antenna serving PSII. The major pigment-proteins of the cyanobacterial PSII antenna are phycobiliproteins, which absorb only in red-orange light in *Synechococcus* 7942 and *Synechocystis* 6803 [40]. Under iron-replete growth both strains thus showed large PSII effective absorbance cross sections for red-orange light (*Synechococcus*, 381 A^2^ PSII^−1^; *Synechocystis* (412 A^2^ PSII^−1^), largely resulting from light absorbed by the phycobiliproteins. There is also a small amount of chlorophyll present in the inner antenna pigment proteins of PSII, which absorbs both red (670 nm) and blue light [41]. As expected, both strains showed small PSII effective absorbance cross sections for blue light (*Synechococcus*, 79 A^2^ PSII^-1^; *Synechocystis*, 71 A^2^ PSII^−1^), commensurate with the low chlorophyll content of PSII. Thus, under our initial conditions of iron replete growth media, the PSII antenna is much more effective at capturing red light than blue light in these cyanobacteria. Küpper [22] proposed that the IsiA chlorophyll-protein associates with PSII to form an energy harvesting complex analogous to the IsiA-PSI complex. IsiA is a chlorophyll binding protein, thus it absorbs both red and blue light. If IsiA associates with PSII as a light capturing molecule, the effective cross section of PSII under blue light should increase relative to red light since IsiA absorbs both red and blue light, whereas the phycobilisome absorbs only red light. To determine if this is the case, we determined the σ_PSII_ values for blue light during our iron starvation treatments. In both strains there was no appreciable increase in either the total effective absorbance cross section under blue light nor in the ratio between blue and red effective absorbance cross sections for PSII (Fig. 3A, B). These observations were consistent across all actinic light levels measured (Fig. 3C, D, E, F). This indicates that under our treatment conditions IsiA does not contribute detectably to the light capture antenna of PSII in either *Synechococcus* nor in *Synechocystis*, even though IsiA accumulates to levels in excess of known IsiA:PSI complexes, at least in *Synechococcus*. This excess IsiA may function to protect PSII from photodamage, as suggested by functional and pigment analyses [23], but we have no evidence that it contributes to excitation capture serving PSII.

### PSII Function and Electron Transport

**Figure 4:**
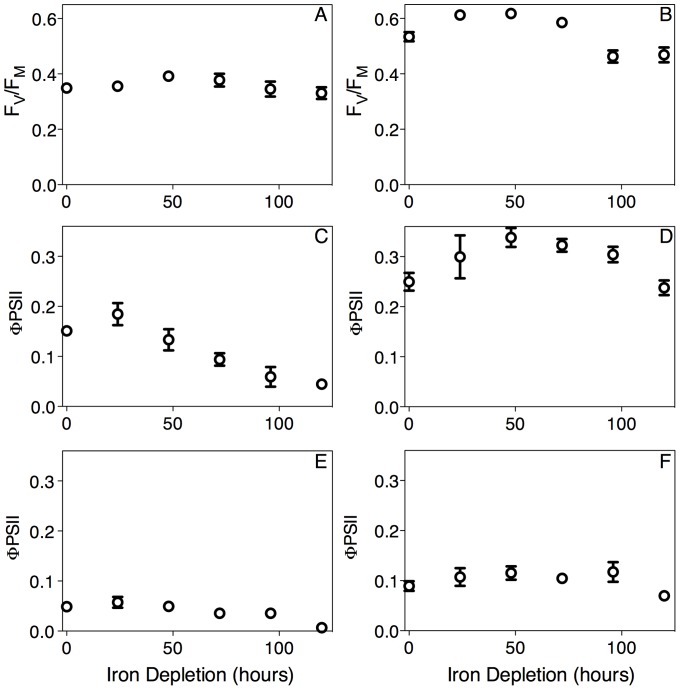
Photosystem II maximum quantum yield (F_v_/F_m_) (A, B) or Photosystem II quantum yield for electron transport (Φ_PSII_) (C, D, E, F) in *Synechococcus* (A, C, E) or *Synechocystis* (B, D, F) over a 120 hour iron depletion timecourse. (A, B) F_v_/F_m_ measured from cells under 0 μmol photons·m^−2^·s^−1^. (C, D) Φ_PSII_ measured from cells under the growth light level of 65 μmol photons·m^−2^·s^−1^. (E, F) Φ_PSII_ measured from cells under saturating light of 262 μmol photons·m^−2^·s^−1^, 4X higher than the growth light level. Data were compiled from 6 (*Synechococcus*) or 5 (*Synechocystis*) replicate measurements from 6 or 5 separate cyanobacterial cultures. All yield data were captured using blue light excitation of fluorescence. Data presented are mean +/− standard error, n = 5 or 6.

In cyanobacteria fluorescence-based estimates of PSII quantum yield must be interpreted with caution because phycobiliproteins [28], PSI [42] and IsiA [16] can contribute to the base line F_O_ fluorescence level [42] to variable extents depending upon the wavelength bands used to excite and detect fluorescence, thereby distorting measures of F_V_/F_M_ and Φ_PSII_. Nevertheless, with caution, these ratios can be interpreted. We used a single turnover flashlet train of blue light of 455 nm to excite fluorescence, which we detected over the wavelength range 695–720 nm, for determination of F_V_/F_M_ and Φ_PSII_. We chose 455 nm excitation to limit excitation of distorting fluorescence from phycobiliproteins [28]. Our 695–720 nm fluorescence emission detection waveband in turn limited the contribution of fluorescence from IsiA [43] to our F_O_ fluorescence. During the iron depletion time course we measured only limited variation in the level of the maximal quantum yield of PSII, F_V_/F_M_, in both *Synechococcus* (Fig. 4A) and *Synechocystis* (Fig. 4B). This is in contrast to the considerable decreases in F_V_/F_M_ for *Synechocystis* reported by Sandstrom et al. [6] Schrader et al. [16] and Ryan-Keogh et al. [18], but we think the differences result from the specific excitation and emission wavelength bands, which in our case limited the influence of IsiA fluorescence upon the measured F_V_/F_M_, rather than from any fundamental biological distinction between our study and the results of Sandstrom et al. [6], Schrader et al. [16] and Ryan-Keogh et al. [18]. In general, cyanobacterial F_V_/F_M_ measurements depend sensitively upon the specific excitation and emission wavebands [42], as well as the pigment composition of the cultures.

Plastoquinone reduced by PSII photochemistry donates electrons to cytochrome b_6_f before returning to a PSII complex to receive another electron. If the capacity for electron transport away from PSII is decreased, there will be a bottleneck in electron transport, as the cells cannot transfer electrons fast enough to keep up with the supply from PSII [5]. This will lead to a reduced plastoquinone pool that cannot accept further electrons from PSII, which thus remain in the closed state for longer periods of time. This PSII closure lowers the quantum yield for electron transport (Φ_PSII_) under illumination. Under the growth light level the Φ_PSII_ values for *Synechococcus* decreased significantly below control levels within 72 h of iron depletion (Fig. 4C, p<0.05 from 1 way ANOVA with Dunnett's Multiple Comparison Test). This indicates that a larger proportion of PSII reaction centres were becoming closed at the given growth light level in the iron depleted cultures than in iron replete cultures. The Φ_PSII_ values for *Synechocystis* remained fairly stable during the iron depletion time course (Fig. 4D), likely because the cells were not as far into iron starvation as *Synechococcus*, or because the electron transport away from PSII flows through alternative pathways in this strain [2]. Under saturating light levels (Fig. 4E, F) Φ_PSII_ was suppressed to low levels for both *Synechococcus* and *Synechocystis* across the iron depletion time course.

Electron transport away from PSII can be estimated by multiplying σ_PSII_', the effective absorbance cross section serving PSII photochemistry, by incident photons per area per unit time, and by q_P_, the proportion of PSII instantaneously open and ready to perform photochemistry, to give electrons transported per unit time per PSII [26].

**Figure 5:**
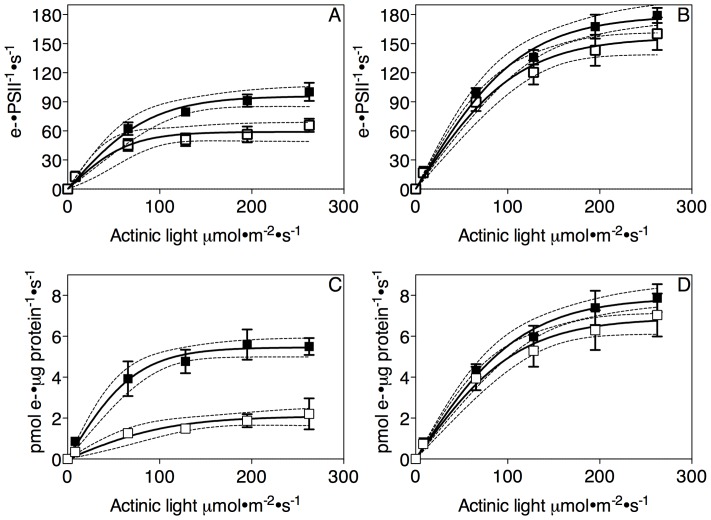
Light response curves for electron transport per PSII (A, B) or per total cellular protein (C, D) in *Synechococcus* (A, C) or *Synechocystis* (B, D) after 0 (closed symbols) or 120 hours (open symbols) of iron depletion. Data are expressed in electrons per PSII per second (A, B) or in pmol electrons per μg protein per s. Data were compiled from 6 (*Synechococcus*) or 5 (*Synechocystis*) replicate measurements of σ_PSII_ and q_P_ performed on 6 or 5 separate cyanobacterial cultures. For estimation of pmol electrons per μg protein per s we also approximated PSII content as fmol PsbA ug total protein^−1^. Curve fits are photosynthesis/irradiance curves with 95% confidence intervals plotted as outer dotted lines. Iron depletion led significant changes in the curve fits.

Electron transport per PSII in *Synechococcus* (Fig. 5A) declined significantly by the end of the iron depletion trial. A hyperbolic tangent (Michaelis-Menten) fit of electron transport versus irradiance shows that Pmax declined from 96 (±4, SEM) to 59 (±3, SEM) e- PSII^−1^ s^−1^ over 120 h of iron depletion. In parallel the E_K_ light saturation parameter declined from 90 to 65 μmol photons•m^−2^•s^−1^ over the iron depletion time course. Electron transport per PSII also declined somewhat in *Synechocystis* cells but only from 179 (±7, SEM) to 157 (±7, SEM) e- PSII^−1^ s^−1^, which provides further evidence that this strain did not progress as far into iron depletion as did *Synechococcus*. By multiplying electron transport per PSII [26] by the estimated PSII content from the PsbA and PsbD protein subunit data, we estimated PSII electron transport s^−1^ μg protein^−1^, subject to the caveat that not all PSII protein subunits are part of active PSII centers, since some must be engaged in the PSII repair cycle at any given time. This estimate decreased substantially in *Synechococcus* (Fig. 5C) and marginally in *Synechocystis* (Fig. 5D) after 120 h of iron depletion. In the case of *Synechococcus* the drop in electron transport per PSII is compounded by the drop in PSII content per total protein (Fig. 2G, I), to generate a large drop in total electron transport from the PSII pool.

Achieved electron transport is dependent upon numerous complexes and molecules downstream from PSII [44]. Cytochrome b_6_f is the complex responsible for taking electrons from the plastoquinone pool and donating them to plastocyanin on their way to PSI in photosynthetic electron transport [35]. Because it is such an iron-expensive complex to synthesize, in times of iron scarcity it is synthesized in lower quantities [4]–[6]. In both *Synechococcus* and *Synechocystis* the downward trends in both Cytochrome b_6_f and PSI abundance (Figs. 1C, D, E, F) contribute an explanation for the decrease in PSII electron transport, although the decrease in Cytochrome b_6_f was not as pronounced in *Synechococcus* which showed the greater decrease in electron transport away from PSII. As there was a greater decrease in PSI content in *Synechococcus* than there was in *Synechocystis* it is likely that the sharp decline in PSI:PSII ratio explains the greater decrease in cellular electron transport in *Synechococcus*.

Electron transport per PSII, was higher in *Synechocystis* compared to *Synechococcus* both before and after iron starvation, even though in iron replete conditions there were higher amounts of both PSI and cytochrome b_6_f in *Synechococcus*. There were similar amounts of PSII in the strains both in terms of fmol/μg and percentage of total protein, indicating a similar capacity to pump electrons into electron transport. This indicates that there were other important electron transport complexes or electron sinks along the way [2], [3], [5], [44] that helped oxidize the *Synechocystis* electron transport pathway, allowing PSII to continue pumping electrons at a high rate [4]. This theory is also supported by the Φ_PSII_ values for *Synechocystis* which do not substantially decrease as iron starvation goes on.

### Changing chlorophyll allocations under iron depletion

**Figure 6:**
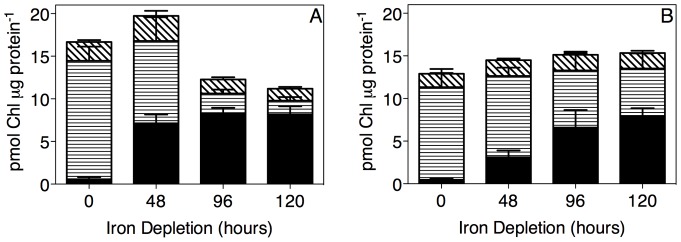
Chlorophyll allocations in *Synechococcus* (A) and *Synechocystis* (B) over a 120 hour iron depletion timecourse. Data were compiled from 6 (*Synechococcus*) or 5 (*Synechocystis*) replicate time course experiments. (A, B) Solid bars: IsiA subunit contents per μg of total cellular protein (Figure 1) were multiplied by 12 chlorophyll bound per IsiA monomer; Horizontal cross-hatching: PsaC subunit contents per μg of total cellular protein (Figure 1) were multiplied by 100 chlorophyll bound per PSI monomer; Diagonal cross-hatching: PsbA subunit contents per μg of total cellular protein (Figure 1) were multiplied by 36 per PSII monomer. Data presented are mean +/− standard error, n = 5 or 6.

Figure 6 presents estimates of the allocations of cellular chlorophyll among the major binding complexes of IsiA (solid bars), PSI (horizontal cross-hatching) and PSII (diagonal cross-hatching). In iron-replete cells of both *Synechococcus* (6A) and *Synechocystis* (6B), most chlorophyll is bound to PSI complexes, as expected from the 100 chlorophyll stoichiometry per PSI complex compared to only 36 chlorophyll per PSII, and the ∼2.3 to 2.5:1 ratio of PSI:PSII.

Over the initial 48 h of iron depletion *Synechococcus* shows a near 1 for 1 exchange of PSI chlorophyll to IsiA chlorophyll, as PSI content drops and IsiA accumulates (Fig. 6A). Thereafter, levels of chlorophyll bound to IsiA saturates as IsiA accumulation reaches a maximum (Fig. 2A), while PSI chlorophyll continues to drop, leading to a drop in overall chlorophyll per protein, a pattern reflected in our absorbance estimates for chlorophyll per cell (Fig. 1E) for *Synechococcus*.

In *Synechocystis* the shift from PSI chlorophyll to IsiA chlorophyll progressed over the 120 h time course of iron depletion, with no evidence for a drop in total chlorophyll per total protein (Fig. 6B), even though total chlorophyll absorbance per cell (Fig. 1F) declined somewhat. The chlorophyll allocation pattern may reflect the slower progression of the *Synechocystis* acclimation to iron depletion, since at 96 to120 h the chlorophyll allocation was qualitatively similar to the pattern for *Synechoccocus* after 48 h. The *Synechococccus* data indicate that ∼690 fmol IsiA μg protein^−1^ is an upper limit for the accumulation of this chlorophyll-binding protein, and once reached, further drops in PSI content lead to loss of chlorophyll per cell. We suspect that *Synechocystis* would show a similar saturation of IsiA chlorophyll accumulation upon more prolonged exposure to iron depletion. The strain specific differences in the timing of these compositional acclimations to iron depletion are not attributable to a difference in growth rate, since the cultures grew at similar rates over the first 72 h of iron depletion (Figs. 1A, B). *Synechococcus* and *Synechocystis* share conserved photosynthetic complexes, and both induce IsiA upon iron depletion, but the regulatory timing of the acclimatory processes is distinct.

The results presented here provide new insights into the mechanisms of IsiA mediation of and other photosynthetic adaptations to iron depletion in two different model cyanobacterial species. While IsiA has been shown previously to increase the functional absorption cross-section of PSI [8], [9], [18], we show that it is not acting in a similar fashion for PSII. Rather it seems that IsiA accumulates beyond levels able to serve in light capture for PSI, forming structures for storage of chlorophyll with safe dissipation of absorbed excitation [23] which may allow these organisms to more quickly recover when iron becomes available again without the risk of photodamage during the iron starvation period. Our quantitative immunoblotting results furthermore show considerable differences in the stoichiometries of the photosynthetic apparatus of these two species both under normal growth conditions and in response to iron starvation.

## References

[1] Peschek GA, Obinger C, Sherman DM, Sherman LA (1994) Immunocytochemical localization of the cytochrome-c oxidase in a cyanobacterium, *Synechococcus* PCC7942 (*Anacystis nidulans*). BBA-Bioenergetics 1187: 369–372.

[2] Peschek GA, Obinger C, Fromwald S, Bergman B (1994) Correlation between immuno-gold labels and activities of the cytochrome-c oxidase (aa3-type) in membranes of salt stressed cyanobactria. FEMS Microbiol Lett 124: 431–437 doi:10.1111/j.1574-6968.1994.tb07320.x.

[3] Schubert H, Matthijs HCP, Mur LR (1995) In vivo assay of P700 redox changes in the cyanobacterium *Fremyella diplosiphon* and the role of cytochrome c oxidase in regulation of photosynthetic electron transfer. Photosynthetica 31: 517–527.

[4] Bailey S, Melis A, Mackey KRM, Cardol P, Finazzi G, et al (2008) Alternative photosynthetic electron flow to oxygen in marine *Synechococcus* . Biochim Biophys Acta 1777: 269–276 doi:10.1016/j.bbabio.2008.01.002. 18241667 10.1016/j.bbabio.2008.01.002

[5] Ivanov AG, Park YI, Miskiewicz E, Raven JA, Huner NP, et al (2000) Iron stress restricts photosynthetic intersystem electron transport in *Synechococcus* sp. PCC 7942. FEBS Lett 485: 173–177. 11094162 10.1016/s0014-5793(00)02211-0

[6] Sandström S, Ivanov AG, Park Y-I, Oquist G, Gustafsson P (2002) Iron stress responses in the cyanobacterium *Synechococcus* sp. PCC7942. Physiol Plant 116: 255–263. 12354203 10.1034/j.1399-3054.2002.1160216.x

[7] Dijkman NA, Kroon BMA (2002) Indications for chlororespiration in relation to light regime in the marine diatom *Thalassiosira weissflogii* . J Photochem Photobiol B Biol 66: 179–187. 10.1016/s1011-1344(02)00236-1 11960727

[8] Kouril R, Arteni AA, Lax J, Yeremenko N, D'Haene S, et al (2005) Structure and functional role of supercomplexes of IsiA and Photosystem I in cyanobacterial photosynthesis. FEBS Lett 579: 3253–3257 doi:10.1016/j.febslet.2005.03.051. 15943969 10.1016/j.febslet.2005.03.051

[9] Ivanov AG, Krol M, Sveshnikov D, Selstam E, Sandström S, et al (2006) Iron deficiency in cyanobacteria causes monomerization of Photosystem I trimers and reduces the capacity for state transitions and the effective absorption cross section of Photosystem I in vivo. Plant Physiol 141: 1436–1445 doi:10.1104/106.082339. 16798943 10.1104/pp.106.082339 PMC1533926

[10] Havaux M, Guedeney G, Hagemann M, Yeremenko N, Matthijs HCP, et al (2005) The chlorophyll-binding protein IsiA is inducible by high light and protects the cyanobacterium *Synechocystis* PCC6803 from photooxidative stress. FEBS Lett 579: 2289–2293 doi:10.1016/j.febslet.2005.03.021. 15848160 10.1016/j.febslet.2005.03.021

[11] Leonhardt K, Straus NA (1992) An iron stress operon involved in photosynthetic electron transport in the marine cyanobacterium *Synechococcus* sp. PCC 7002. J Gen Microbiol 138 Pt 8: 1613–1621. 10.1099/00221287-138-8-1613 1527503

[12] Rueter JG, Ohki K, Fujita Y (1990) The Effect of Iron Nutrition on Photosynthesis and Nitrogen Fixation in Cultures of Trichodesmium (cyanophyceae)1. Journal of Phycology 26: 30–35 doi:10.1111/j.0022-3646.1990.00030.x.

[13] Shi T, Sun Y, Falkowski PG (2007) Effects of iron limitation on the expression of metabolic genes in the marine cyanobacterium Trichodesmium erythraeum IMS101. Environmental Microbiology 9: 2945–2956 doi:10.1111/j.1462-2920.2007.01406.x. 17991025 10.1111/j.1462-2920.2007.01406.x

[14] Richier S, Macey AI, Pratt NJ, Honey DJ, Moore CM, et al (2012) Abundances of Iron-Binding Photosynthetic and Nitrogen-Fixing Proteins of Trichodesmium Both in Culture and In Situ from the North Atlantic. PLoS ONE 7: e35571 doi:10.1371/journal.pone.0035571. 22563465 10.1371/journal.pone.0035571 PMC3341377

[15] Grossman AR, Schaefer MR, Chiang GG, Collier JL (1993) The phycobilisome, a light-harvesting complex responsive to environmental conditions. Microbiol Rev 57: 725–749. 8246846 10.1128/mr.57.3.725-749.1993 PMC372933

[16] Schrader PS, Milligan AJ, Behrenfeld MJ (2011) Surplus photosynthetic antennae complexes underlie diagnostics of iron limitation in a cyanobacterium. PLoS ONE 6: e18753 doi:10.1371/journal.pone.0018753. 21533084 10.1371/journal.pone.0018753 PMC3080375

[17] Aspinwall CL, Duncan J, Bibby T, Mullineaux CW, Barber J (2004) The trimeric organisation of Photosystem I is not necessary for the iron-stress induced CP43′ protein to functionally associate with this reaction centre. FEBS Lett 574: 126–130 doi:10.1016/j.febslet.2004.08.016. 15358552 10.1016/j.febslet.2004.08.016

[18] Ryan-Keogh TJ, Macey AI, Cockshutt AM, Moore CM, Bibby TS (2012) The cyanobacterial chlorophyll-binding-protein IsiA acts to increase the in vivo effective absorption cross-section of PSI under iron limitation. J phycol 48: 145–154 doi:10.1111/j.1529-8817.2011.01092.x. 27009659 10.1111/j.1529-8817.2011.01092.x

[19] Melkozernov AN, Bibby TS, Lin S, Barber J, Blankenship RE (2003) Time-resolved absorption and emission show that the CP43′ antenna ring of iron-stressed *Synechocystis* sp. PCC6803 is efficiently coupled to the photosystem I reaction center core. Biochemistry 42: 3893–3903 doi:10.1021/bi026987u. 12667080 10.1021/bi026987u

[20] Chauhan D, Folea IM, Jolley CC, Kouřil R, Lubner CE, et al (2011) A novel photosynthetic strategy for adaptation to low-iron aquatic environments. Biochemistry 50: 686–692 doi:10.1021/bi1009425. 20942381 10.1021/bi1009425

[21] Yeremenko N, Kouril R, Ihalainen JA, D'Haene S, Van Oosterwijk N, et al (2004) Supramolecular organization and dual function of the IsiA chlorophyll-binding protein in cyanobacteria. Biochemistry 43: 10308–10313 doi:10.1021/bi048772l. 15301529 10.1021/bi048772l

[22] Küpper H, Setlík I, Seibert S, Prásil O, Setlikova E, et al (2008) Iron limitation in the marine cyanobacterium *Trichodesmium* reveals new insights into regulation of photosynthesis and nitrogen fixation. New Phytol 179: 784–798 doi:10.1111/j.1469-8137.2008.02497.x. 18513224 10.1111/j.1469-8137.2008.02497.x

[23] Ivanov AG, Krol M, Selstam E, Sane PV, Sveshnikov D, et al (2007) The induction of CP43′ by iron-stress in *Synechococcus* sp. PCC 7942 is associated with carotenoid accumulation and enhanced fatty acid unsaturation. Biochim Biophys Acta 1767: 807–813 doi:10.1016/j.bbabio.2007.02.006. 17362874 10.1016/j.bbabio.2007.02.006

[24] Rippka R, Deruelles J, Waterbury JB, Herdman M, Stanier RY (1979) Generic assignments, strain histories and properties of pure cultures of cyanobacteria. J Gen Microbiol 111: 1–61 doi:10.1099/00221287-111-1-1.

[25] Kolber, Prášil O, Falkowski B (1998) Measurements of variable chlorophyll fluorescence using fast repetition rate techniques: defining methodology and experimental protocols. Biochim Biophys Acta 1367: 88–106. 9784616 10.1016/s0005-2728(98)00135-2

[26] Huot Y, Babin M (2010) Overview of fluorescence protocols: theory, basic concepts, and practice. In: Suggett DJ, Prášil O, Borowitzka MA, editors. Chlorophyll *a* Fluorescence in Aquatic Sciences: Methods and Applications. Dordrecht: Springer Netherlands. 31–74. Available: http://www.springerlink.com/index/10.1007/978-90-481-9268-7_3. Accessed 2012 Oct 29.

[27] Van Kooten O, Snel JFH (1990) The use of chlorophyll fluorescence nomenclature in plant stress physiology. Photosynthesis Research 25: 147–150. 24420345 10.1007/BF00033156

[28] Campbell D, Hurry V, Clarke AK, Gustafsson P, Öquist G (1998) Chlorophyll fluorescence analysis of cyanobacterial photosynthesis and acclimation. Microbiol Mol Biol Rev 62: 667–683. 9729605 10.1128/mmbr.62.3.667-683.1998 PMC98930

[29] Burns RA, Mac Kenzie TD, Campbell DA (2006) Inorganic carbon repletion constrains steady-state light acclimation in the cyanobacterium *Synechococcus elongatus* . J Phycol 42: 610–621.

[30] Brown C, MacKinnon J, Cockshutt A, Villareal T, Campbell D (2008) Flux capacities and acclimation costs in *Trichodesmium* from the Gulf of Mexico. Mar Biol 154: 413–422 doi:10.1007/s00227-008-0933-z.

[31] Ferreira KN, Iverson TM, Maghlaoui K, Barber J, Iwata S (2004) Architecture of the photosynthetic oxygen-evolving center. Science 303: 1831–1838 doi:10.1126/science.1093087. 14764885 10.1126/science.1093087

[32] Murray JW, Duncan J, Barber J (2006) CP43-like chlorophyll binding proteins: structural and evolutionary implications. Trends Plant Sci 11: 152–158 doi:10.1016/j.tplants.2006.01.007. 16473546 10.1016/j.tplants.2006.01.007

[33] Öquist G (1971) Changes in pigment composition and photosynthesis induced by iron-deficiency in the blue-green alga *Anacystis nidulans* . Physiol Plant 25: 188–191 doi:10.1111/j.1399-3054.1971.tb01426.x.

[34] Fromme P (1996) Structure and function of Photosystem I. Curr Opin Struct Biol. 6: 473–484 doi:10.1016/S0959-440X(96)80112-6. 10.1016/s0959-440x(96)80112-6 8794163

[35] Vermaas WF (2001) Photosynthesis and respiration in cyanobacteria. In: John Wiley & Sons, Ltd, editor. Encyclopedia of Life Sciences. Chichester, UK: John Wiley & Sons, Ltd. 1–7. Available:http://www.els.net/WileyCDA/ElsArticle/refId-a0001670.html. Accessed 2012 Oct 29.

[36] Barber J, Kühlbrandt W (1999) Photosystem II. Curr Opin Struct Biol 9: 469–475 doi:10.1016/S0959-440X(99)80066-9. 10449373 10.1016/S0959-440X(99)80066-9

[37] Nixon PJ, Michoux F, Yu J, Boehm M, Komenda J (2010) Recent advances in understanding the assembly and repair of Photosystem II. Ann Botany 106: 1–16 doi:10.1093/aob/mcq059. 20338950 10.1093/aob/mcq059 PMC2889791

[38] Yao DCI, Brune DC, Vavilin D, Vermaas WFJ (2012) Photosystem II component lifetimes in the cyanobacterium *Synechocystis* sp. strain PCC 6803: small Cab-like proteins stabilize biosynthesis intermediates and affect early steps in chlorophyll synthesis. J Biol Chem 287: 682–692 doi:10.1074/jbc.M111.320994. 22090028 10.1074/jbc.M111.320994 PMC3249123

[39] Stock D, Gibbons C, Arechaga I, Leslie AG, Walker JE (2000) The rotary mechanism of ATP synthase. Curr Opin Struct Biol 10: 672–679 doi:10.1016/S0959-440X(00)00147-0. 11114504 10.1016/s0959-440x(00)00147-0

[40] Yokono M, Murakami A, Akimoto S (2011) Excitation energy transfer between Photosystem II and Photosystem I in red algae: larger amounts of phycobilisome enhance spillover. Biochim Biophys Acta 1807: 847–853 doi:10.1016/j.bbabio.2011.03.014. 21496452 10.1016/j.bbabio.2011.03.014

[41] Schagerl M, Müller B (2006) Acclimation of chlorophyll *a* and carotenoid levels to different irradiances in four freshwater cyanobacteria. J Plant Physiol 163: 709–716 doi:10.1016/j.jplph.2005.09.015. 16325961 10.1016/j.jplph.2005.09.015

[42] Simis SGH, Huot Y, Babin M, Seppälä J, Metsamaa L (2012) Optimization of variable fluorescence measurements of phytoplankton communities with cyanobacteria. Photosynth Res 112: 13–30 doi:10.1007/s11120-012-9729-6. 22403036 10.1007/s11120-012-9729-6 PMC3324691

[43] Joshua S, Bailey S, Mann NH, Mullineaux CW (2005) Involvement of phycobilisome diffusion in energy quenching in cyanobacteria. Plant Physiol 138: 1577–1585 doi:10.1104/105.061168. 15908597 10.1104/pp.105.061168 PMC1176427

[44] Ivanov AG, Sane PV, Simidjiev I, Park Y-I, Huner NPA, et al (2012) Restricted capacity for PSI-dependent cyclic electron flow in ΔpetE mutant compromises the ability for acclimation to iron stress in *Synechococcus* sp. PCC 7942 cells. Biochim Biophys Acta 1817: 1277–1284 doi:10.1016/j.bbabio.2012.03.014. 22465025 10.1016/j.bbabio.2012.03.014

